# Testing Protein Leverage in Lean Humans: A Randomised Controlled Experimental Study

**DOI:** 10.1371/journal.pone.0025929

**Published:** 2011-10-12

**Authors:** Alison K. Gosby, Arthur D. Conigrave, Namson S. Lau, Miguel A. Iglesias, Rosemary M. Hall, Susan A. Jebb, Jennie Brand-Miller, Ian D. Caterson, David Raubenheimer, Stephen J. Simpson

**Affiliations:** 1 School of Biological Sciences, The University of Sydney, Sydney, Australia; 2 School of Molecular Bioscience, The University of Sydney, Sydney, Australia; 3 Boden Institute of Obesity, Nutrition and Exercise, University of Sydney, Sydney, Australia; 4 Medical Research Council, Human Nutrition Research, Cambridge, United Kingdom; 5 Institute for Natural Sciences, Massey University, Auckland, New Zealand; Pennington Biomedical Research Center, United States of America

## Abstract

A significant contributor to the rising rates of human obesity is an increase in energy intake. The ‘protein leverage hypothesis’ proposes that a dominant appetite for protein in conjunction with a decline in the ratio of protein to fat and carbohydrate in the diet drives excess energy intake and could therefore promote the development of obesity. Our aim was to test the ‘protein leverage hypothesis’ in lean humans by disguising the macronutrient composition of foods offered to subjects under *ad libitum* feeding conditions. Energy intakes and hunger ratings were measured for 22 lean subjects studied over three 4-day periods of in-house dietary manipulation. Subjects were restricted to fixed menus in random order comprising 28 foods designed to be similar in palatability, availability, variety and sensory quality and providing 10%, 15% or 25% energy as protein. Nutrient and energy intake was calculated as the product of the amount of each food eaten and its composition. Lowering the percent protein of the diet from 15% to 10% resulted in higher (+12±4.5%, p = 0.02) total energy intake, predominantly from savoury-flavoured foods available between meals. This increased energy intake was not sufficient to maintain protein intake constant, indicating that protein leverage is incomplete. Urinary urea on the 10% and 15% protein diets did not differ statistically, nor did they differ from habitual values prior to the study. In contrast, increasing protein from 15% to 25% did not alter energy intake. On the fourth day of the trial, however, there was a greater increase in the hunger score between 1–2 h after the 10% protein breakfast versus the 25% protein breakfast (1.6±0.4 *vs* 25%: 0.5±0.3, p = 0.005). In our study population a change in the nutritional environment that dilutes dietary protein with carbohydrate and fat promotes overconsumption, enhancing the risk for potential weight gain.

## Introduction

Increased energy intake is a significant contributor to the rising rates of human obesity [1] and an important priority is thus to understand the factors underlying this shift. It has been proposed that a change in the ratio of protein to fat and carbohydrate in the diet may play a central role in increased energy intake – the ‘protein leverage hypothesis’ (PLH) [2], [3]. The role of dietary protein in the emerging obesity epidemic has, however, until recently largely been ignored. This is partly because protein provides only a minor component of the dietary energy for humans (typically around 15%) and also because its intake has remained far more constant over time and across populations than either fat or carbohydrate [2], [4]. However, rather than indicating that protein has played little role in the rising prevalence of obesity over recent decades, the relative constancy of protein intake may, in fact, offer a key to understanding the dietary causes of excess energy intake and obesity [2]. Simpson and Raubenheimer [2] used data from the FAOSTAT [5] nutrient-supply database to show that an estimated decrease in percent dietary protein from 14% to 12.5% between 1961 and 2000 in the USA was associated with a 14% increase in non-protein energy intake, with absolute protein intake remaining almost constant. A recent analysis of The National Health and Nutrition Examination Survey are consistent with this conclusion, showing that a drop in percent dietary protein across the period from 1971 to 2006 has been associated with an increase in total energy intake [6]. Experimental data suggest that the response of humans when faced with imbalanced diets is to prioritize the absolute intake of protein to a ‘target’ level at the expense of regulating fat and carbohydrate intake [2], [3], [6], [7]. Such ‘protein leverage’ [2] has been demonstrated in numerous other species, including non-human primates [8], pigs [9], [10], rodents [10], [11], birds [12], fish [13] and insects [14]. The strength of protein regulation (i.e. the extent of protein leverage) varies between species, but in all these animals when the percentage of protein in the diet is lowered, total energy intake increases in an effort to maintain constant protein intake. The most extreme example of protein leverage reported to date comes from free-ranging spider monkeys [8], in which protein intake was maintained constant across a wide range of % protein diets; whereas in mice protein compensation is partial due to counterbalancing feedbacks from carbohydrate [11].

If the PLH is true for humans, the implications are substantial: a shift towards dilution of protein in the diet by fat and carbohydrate encouraged by economic pressures [15], increased reliance on cheap fats and sugar, and an ancestral tendency to find fat and sugar highly palatable [16], [17] will drive excess energy intake. This will be exacerbated by reduced energy expenditure without a commensurate increase in the proportion of protein in the diet [2]. Excess energy intake predisposes towards obesity which in turn instigates a vicious metabolic cycle, whereby elevated circulating levels of free fatty acids and developing insulin resistance disinhibit protein catabolism and hepatic gluconeogenesis, requiring increased protein intake to maintain muscle mass and amino acid pools. This then drives over-consumption of low-protein diets [2]. Consistent with this, attempts to lose weight are impeded by a reduction in percent dietary protein [7].

Our aim in the present study was to test the predictions of the PLH while controlling for two key confounding factors inherent in previous studies; that changing percent dietary protein typically involves concurrent changes in both food palatability and variety [2], [18]–[21]. Accordingly, we have used recently developed protocols [22] to disguise the macronutrient composition of foods offered to lean subjects, anticipated to have effective appetite regulatory systems. The foods were provided under *ad libitum* feeding conditions and we then measured the effect of manipulating macronutrient balance on energy intake.

## Results

### Intake

**Figure 1:**
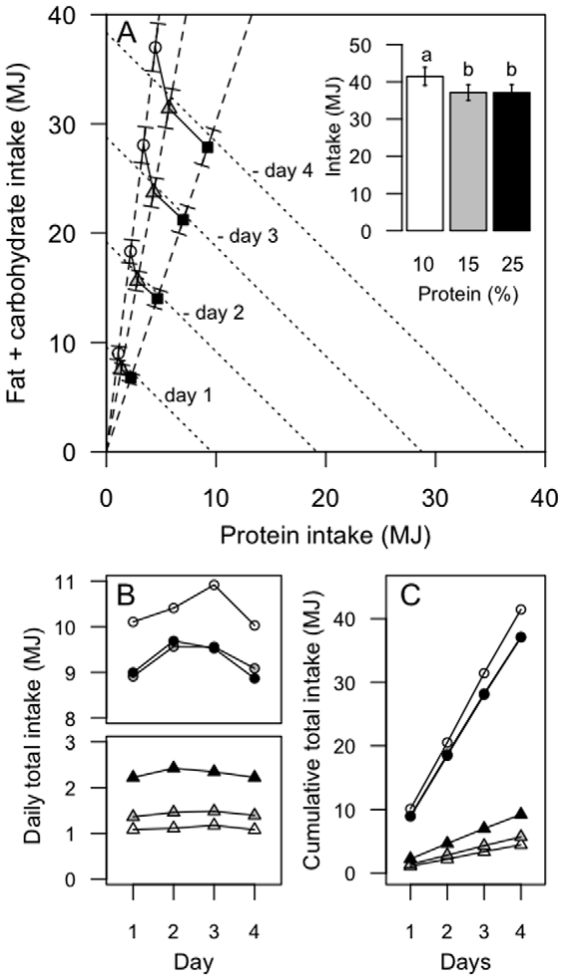
Lean humans increase energy consumption on a lower percent protein diet. (**A**) Cumulative daily bi-coordinate means for protein and non-protein macronutrient (carbohydrate and fat) intake (MJ) for participants during the 4-day 10% (white circles), 15% (grey triangles) and 25% (black squares) *ad libitum* study periods. The dashed lines represent the nutrient rails participants were restricted to during the 10%, 15% and 25% study periods. The dotted lines represent intakes that may occur on the 10%, 15% and 25% foods if intake was regulated to energy requirements (calculated as 1.6× basal energy requirements as derived from the Schofields equation [23] assuming a light to moderate level of physical activity) and that protein and carbohydrate were interchangeable. The inset shows total energy intake (MJ) for participants over the 4-day 10% (white), 15% (grey) and 25% (black) *ad libitum* study periods. The same letter above the bars in the insert indicates that the means did not differ significantly in Bonferroni post hoc comparisons, whereas different letters indicate differences at p<0.05. Refer to Figure S1 for individual total energy intake data points. (**B**) Daily protein (triangles) and total (circles) intake (MJ) for participants during the 4-day 10% (white), 15% (grey) and 25% (black) *ad libitum* study periods. Intake did not change statistically across days within each treatment. Note that the appearance of an increase in intake (of all nutrients, due to fixed diet compositions) from days 1 to 2 and a decline from day 3 to 4 reflected commencement of the study after breakfast on day 1 and fasting overnight on day 4 in readiness for a meal test on day 5 (data not reported). (**C**) Cumulative protein (triangles) and total (circles) intake (MJ) for participants during the 4-day 10% (white), 15% (grey) and 25% (black) *ad libitum* study periods.

Participants consumed an average of 4.34 MJ more energy (a 12% increase) over the 4-day period on a 10% protein diet than on a 15% protein diet (P_10%*vs*15%_<0.0001; Table 1, Figure 1A; refer to Figure S1 for individual data points). This increased energy intake on the 10% diet was the net result of eating 1.24 MJ less protein energy (a decrease of 3% total energy) and 5.59 MJ more carbohydrate and fat (an increase of 15% total energy). Participants consumed 1.73±0.08 (10% P), 1.55±0.07 (15% P) and 1.54±0.07 (25%P) times their predicted basal energy requirements based on the Schofield equation, consistent with a light to moderate level of physical activity [23]. That subjects were close to metabolic equilibrium was indicated by estimated habitual protein intakes being similar to protein intakes during the 10% and 15% treatment periods: there was no difference in total urinary urea excretion prior to and following the 10% (p = 0.1) and 15% (p = 0.6) protein study periods (Figure S2). Habitual percent dietary protein was estimated to be approximately 18.6±.0.9, 18.3±1.1 and 18.7±0.7 prior to each 10, 15 and 25% study period. The significantly increased total energy intake on the lower-protein diet was evident from the first day of the trial and continued throughout the subsequent 3 days (Figure 1B). Daily protein and energy intakes were constant and the cumulative increase in energy intake on the 10% protein diet remained significant from day-1 through to day-4 of the trial (Figure 1C). There was no effect of order in which the three treatment regimes (10, 15 or 25% protein) were experienced (F(_2, 42_) = 0.5, P = 0.6). Fibre, salt and sugar (expressed as percent of intake, by weight) (Table 2) did not correlate with energy intake suggesting that changes in percentage of these nutrients did not play a significant role in driving increased energy intakes (Table 3).

**Table 1:** Total energy and nutrient intakes over the 4-day 10%, 15% and 25% protein *ad-libitum* study periods.

	10%	15%	25%	df	F-value	P-value
**energy (MJ)**	41.45±2.43	37.11±2.08†	37.07±2.16†	2,42	6.73	0.002*
**protein (MJ)**	4.46±0.25	5.70±0.31†	9.23±0.53† ‡	2,42	125.56	<0.0001*
**non-protein (MJ)**	37.00±2.19	31.41±1.78†	27.84±1.63† ‡	2.42	29.17	<0.0001*
**fat (MJ)**	12.00±0.72	10.77±0.62†	10.87±0.64†	2,42	5.97	0.004*
**carbohydrate (MJ)**	24.99±1.47	20.64±1.16†	16.97±0.98† ‡	2,42	43.37	<0.0001*
**sugars (g)**	449±26	469±26	468±28	2,42	0.63	0.5
**fibre (g)**	97±6	85±5†	79±4† ‡	2,42	19.39	<0.0001
**salt (g)**	12.0±0.8	11.9±0.8	13.3±0.8† ‡	2,42	8.96	0.0006

Values are means ± SEM. A one-way within subject ANOVA was used to determine differences between the 10%, 15% and 25% protein study periods.

*represents Greenhouse-Geiser corrected P-values due to violation of sphericity assumption. Post-hoc analysis was performed using paired t-test with Bonferroni correction.

† compared to 10%;

‡ compared to 15%.

**Table 2:** Percent fibre, sugar and salt intakes over each 4-day *ad libitum* periods.

					ANOVA	
	10%	15%	25%	df	F-value	P-value
**fibre (%)**	1.57±0.03	1.50±0.03	1.37±0.03† ‡	2,42	37.2	<0.0001
**sugar (%)**	7.3±0.2	8.3±0.2†	8.0±0.2†	2,42	12.9	<0.0001
**salt (%)**	0.20±0.04	0.21±0.01†	0.23±0.01† ‡	2,42	19.9	<0.0001

Fibre, sugar and salt intakes expressed as a percent of weight of food eaten. Values are means ± SEM. A one-way within subject ANOVA was used to determine differences between the 10%, 15% and 25% protein study periods. Post-hoc analysis was performed using paired t-test with Bonferroni correction.

† P<0.05 compared to 10%;

‡ P<0.05 compared to 15%.

**Table 3:** Effect of percent fibre, sugar and salt intakes on total energy intake.

	Pearsons	df	t	P-value
**fibre (%)**	−0.14	1, 64	−1.1	0.26
**sugar (%)**	−0.18	1, 64	−1.5	0.14
**salt (%)**	−0.17	1, 64	−1.4	0.18

Fibre, sugar and salt intakes expressed as a percent of weight of food eaten correlated with total energy intake over the 4-day *ad libitum* period.

Fifty-seven percent of the 4.34 MJ increase in total energy intake between the 15% and 10% protein diets was due to increased intake of savoury foods (P_15%*vs*10%_ = 0.03). Intake of sweet foods contributed the remaining 43% of the increase, but the difference was not statistically significant (P_15%*vs*10%_ = 0.2). More strikingly, 70% of the increase came from foods that were available anytime (P_15%*vs*10%_ = 0.02), with the intake of ‘meal-time’ foods remaining statistically unchanged (P_15%*vs*10%_ = 0.26; 30% of the total energy difference).

With an increase from 15% to 25% protein, participants consumed, on average, 3.53 MJ more protein energy (P_25%*vs*15%_<0.0001) and 3.57 MJ less non-protein energy (P_25%*vs*15%_<0.0001); total energy intake did not differ (P_25%*vs*15%_ = 1.0; Table 1, Figure 1A; refer to Figure S1 for individual data points). Hence, whereas reducing dietary protein from 15% to 10% evoked a significantly increased energy intake, an increase from 15% to 25% protein did not lead to a reduction in energy intake. This was evident from the first day of the trials and daily intake did not differ throughout the subsequent 3 days (Figure 1B).

**Figure 2:**
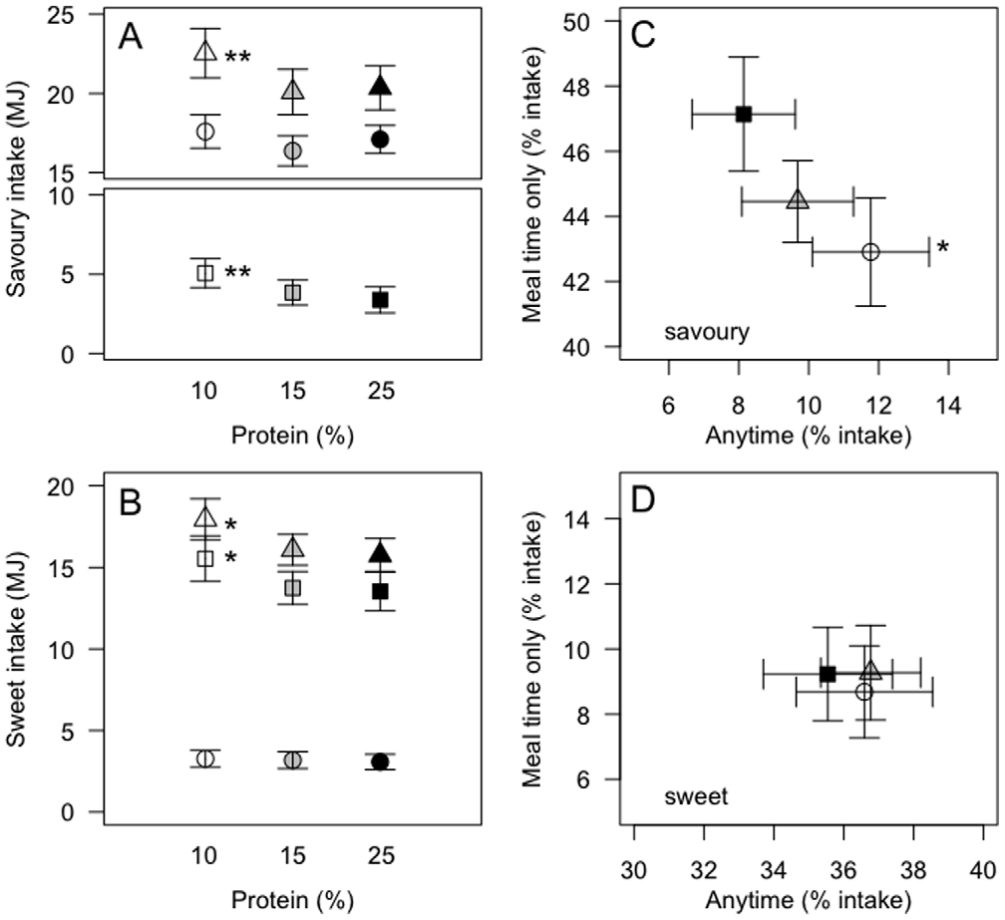
Sweet and savoury intake during the 4-day *ad libitum* period. Total (triangle), anytime (square) and meal-time (circle) savoury (**A**) and sweet (**B**) intake (MJ) during the 4-day *ad libitum* 10% (white), 15% (grey) and 25% (black) protein study periods. Bi-coordinate means for ‘anytime’ and ‘meal time’ savoury (**C**) and sweet (**D**) foods as a percent of total intakes for participants over the 4-day 10% (white circles), 15% (grey triangles) and 25% (black squares) *ad libitum* study periods. Pairwise comparison performed with Bonferroni post hoc comparisons - **10% significantly different to 15% and 25%, p<0.05; * 10% significantly different to 25%, p<0.05.

Although total energy intake during the 15% and 25% protein periods did not differ, the difference in the patterns of intake seen between the 10% and 15% protein periods became more pronounced when comparing the 10% and 25% treatment periods. Thus, increased intake of ‘anytime’ foods contributed 84% of the 4.38 MJ average increase in energy intake between 25% and 10% protein (P_25%*vs*10%_ = 0.0003). Participants significantly increased intake of both sweet (P_25%*vs*10%_ = 0.03) and savoury (P_25%*vs*10%_ = 0.01) foods (Figure 2A and 2B), with each contributing 50% of the total increase. The percent contribution of ‘anytime’ savoury foods was greater during the 10% protein period (P_25%*vs*10%_ = 0.002), and the percent contribution of ‘meal-time’ savoury foods to total intake decreased commensurately (P_25%*vs*10%_ = 0.01) (Figure 2C). The percent contribution of ‘anytime’ (P_25%*vs*10%_ = 0.1) and ‘meal time’ (P_25%*vs*10%_ = 0.7) sweet foods did not differ between the study periods (Figure 2D).

### Subjective hunger and fullness

**Figure 3:**
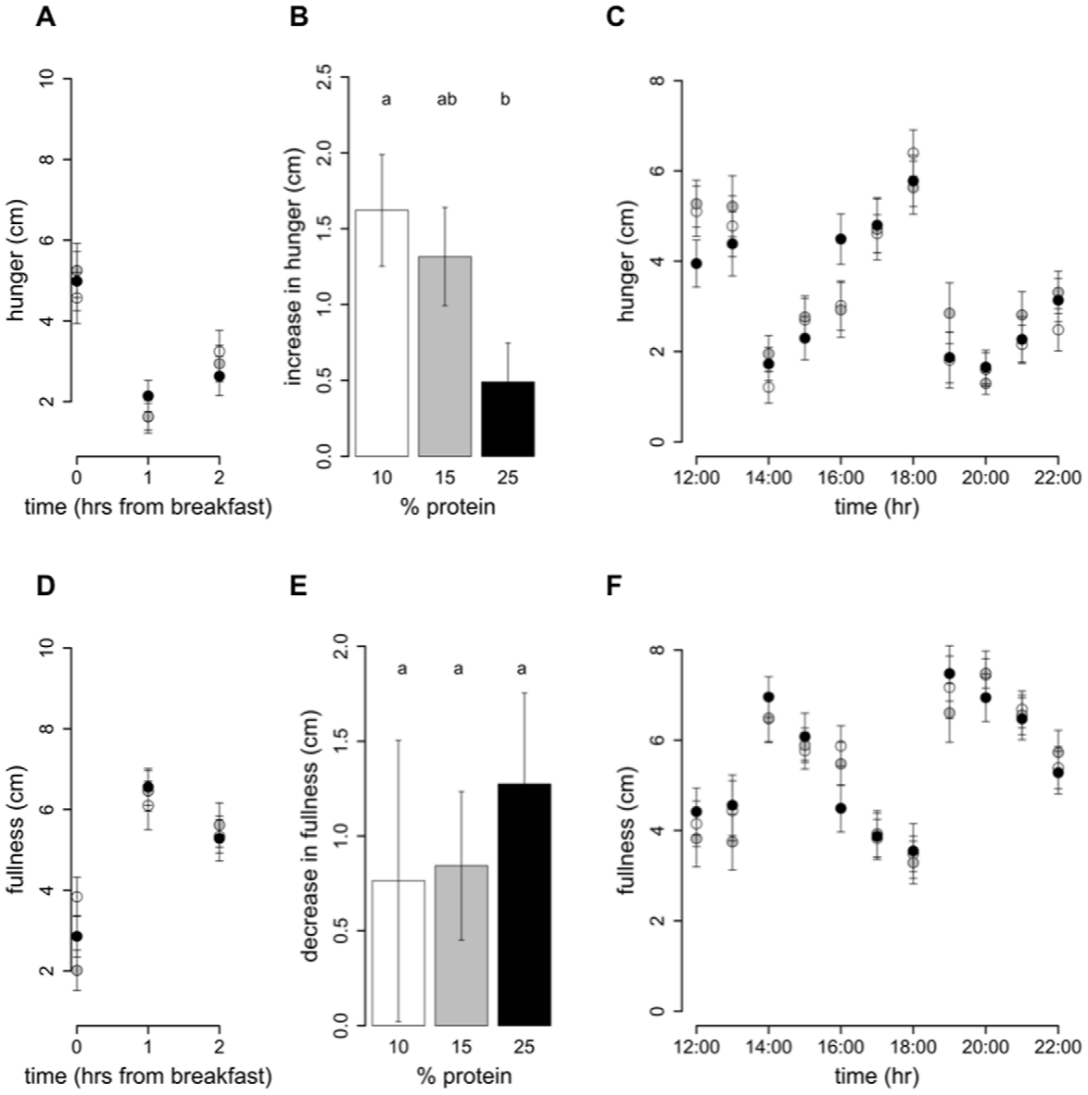
Hunger and fullness scores on 10%, 15% and 25% protein diets. On study day 4, subjects completed a visual analogue scale (VAS) questionnaire to assess subjective hunger and fullness. The VAS questionnaire asked subjects to assess their hunger by reference to a 10 cm horizontal line anchored at one end with the extreme feeling “not at all hungry” and at the other end with “very hungry” and fullness with the extreme feeling “not at all full” and at the other end “very full”. This was done hourly from before breakfast until 10pm. Participants were free to consume breakfast anytime between 08:00–10:00, after which lunch and dinner times were fixed. Hunger and fullness curves have been plotted accordingly. The score prior to breakfast (first of the day) and the 2 scores following breakfast were plotted for hunger (**A**) and fullness (**D**). From 12:00 onwards scores for hunger (**B**) and fullness (**D**) were plotted on the hour. **Figures 3** **C** and **F** show the increase in hunger and decrease in fullness from 1 to 2 h following breakfast. Bars with different letters are significantly different (P<0.05) with Bonferroni post hoc comparisons.

Intake at breakfast did not change with an increase in percent dietary protein from 10% to 25% (P_25%*vs*10%_ = 0.5), and hence protein intake increased (P_25%*vs*10%_<0.0001) and non-protein intake decreased (P_25%*vs*10%_ = 0.001) (Table 4). This is consistent with the finding that *ad libitum* intake of meal-time only foods does not differ with percent dietary protein. Following breakfast the hunger (Figure 3A) and fullness (Figure 3D) scores were unchanged by an increase in percent protein on hunger and fullness ratings taken at 1 h and 2 h with all subjects reporting similarly decreased hunger and increased fullness. However, the increase in hunger from 1 to 2 h was greater following the 10% protein breakfast when compared to the 25% protein breakfast (P_25%*vs*10%_ = 0.005) and a similar trend was evident when protein increased from 15 to 25% (P_25%*vs*15%_ = 0.06) (Figure 3B). In contrast, the decrease in fullness score from 1 to 2 h did not differ with percent protein (P_25%*vs*10%_ = 1.0, P_25%*vs*15%_ = 1.0) (Figure 3E). Figure 3C and F show that hunger and fullness scores did not differ with percent protein from 12:00 onwards. Furthermore, from breakfast (08:00–10:00) until 22:00, hunger and fullness scores did not change with an increase in percent protein from 10 to 15% (hunger: P_15%*vs*10%_ = 0.2 and fullness: P_15%*vs*10%_ = 0.2) or from 15 to 25% (hunger: P_25%*vs*15%_ = 1.0 and fullness: P_25%*vs*10%_ = 1.0) nor was there an effect of percent dietary protein on either the minimum scores (hunger: P_15%*vs*10%_ = 0.7, P_25%*vs*15%_ = 1.0 and fullness: P_15%*vs*10%_ = 0.4, P_25%*vs*15%_ = 0.13) or maximum scores (hunger: P_15%*vs*10%_ = 1.0, P_25%*vs*15%_ = 1.0 and fullness: P_15%*vs*10%_ = 1.0, P_25%*vs*15%_ = 1.0) (Table 5).

**Table 4:** Energy and nutrient intakes at breakfast on day 4 of the 10%, 15% and 25% protein *ad-libitum* study periods.

	10%	15%	25%	df	F-value	P-value
**energy (MJ)**	1.78±0.14	1.74±0.16	1.64±0.14	2, 36	0.8	0.5
**protein (MJ)**	0.18±0.01	0.25±0.02†	0.40±0.03† ‡	2, 36	46.09	<0.0001
**non-protein (MJ)**	1.60±0.12	1.49±0.13†	1.24±0.11†	2, 36	7.5	0.002
**fat (MJ)**	0.53±0.04	0.52±0.05	0.50±0.04	2, 36	0.29	0.8
**carbohydrate (MJ)**	1.07±0.08	0.97±0.09†	0.74±0.06† ‡	2, 36	15.2	<0.0001
**sugars (g)**	25.4±2.5	28.2±2.8	27.8±2.4	2, 36	1.6	0.2
**fibre (g)**	4.6±0.4	5.8±0.5†	4.3±0.4‡	2, 36	14.0	<0.0001
**salt (g)**	0.263±0.023	0.287±0.031	0.355±0.047	2, 36	3.9	0.03

Values are means ± SEM. A one-way within subject ANOVA was used to determine differences between the 10%, 15% and 25% protein study periods. Post-hoc analysis was performed using paired t-test with Bonferroni correction.

† compared to 10%;

‡ compared to 15%.

**Table 5:** Hunger and fullness ratings on day 4 of each of the 10%, 15% and 25% protein study periods.

	10%	15%	25%	df	F-value	P-value
**HUNGER**						
**Mean**	3.2±0.3	3.5±0.3	3.3±0.3	2,36	0.9	0.3
**Lowest**	0.5±0.2	0.6±0.2	0.7±0.2	2,36	1.7	0.2
**highest**	7.3±0.4	7.1±0.5	7.4±0.4	2,36	1.7	0.8
**FULLNESS**						
**Mean**	5.4±0.2	5.1±0.2	5.4±0.3	2,36	0.9	0.4
**Lowest**	1.5±0.3	1.0±0.2	1.7±0.3	2,36	1.9	0.2
**Highest**	9.0±0.2	8.8±0.2	8.5±0.3	2,36	0.9	0.4

Values are means ± SEM. A one-way within subject ANOVA was used to determine differences between the 10%, 15% and 25% protein study periods.

## Discussion

In this randomised, controlled, experimental study we have shown that even when the macronutrient composition of foods was disguised and variety controlled, increased energy intake occurred on diets containing a lower proportion of energy from protein and persisted throughout the four days of the study. This result does not, on its own, demonstrate that protein leverage has been a contributory mechanism to the increased energy intakes that have accompanied the rise in the prevalence of obesity [1] for which we would need to establish that the effect persists over the long-term. However, that the conclusions from our short-term study may also apply in the longer term is supported by two lines of evidence. First, there has been a progressive dilution of protein in the diet over recent decades with associated rises in energy intake and obesity [2], [6]. Second, longer-term experimental trials than ours, in which compliance was ensured but un-disguised foods were used, have shown an association between increased percent dietary protein and a prolonged reduction in total energy intake [7], [18]. If subjects maintained the level of increased intake observed on the 10% protein diet in our study, without an accompanying increase in energy expenditure through increased activity or thermogenesis [24], a 1.0 kg weight increase per month would be expected [25], [26].

Significantly, subjects increased energy intake on the 10% protein diet via increased consumption of ‘anytime foods’, rather than ‘meal time’ foods. Providing constant access to food rather than restricting food to meal times allows subjects to increase the number of eating episodes in a day (‘snacking’ behaviour). This has previously been associated with an increase in total energy intake [27], [28], especially if high energy density foods are chosen [29]. In free-living individuals in the USA the number of eating episodes per day has risen [30]. Increased food variety may also increase total energy intake, as shown in studies that controlled for macronutrient composition [31]. Indeed, evidence suggests that the stimulation of intake by dietary variety is important for achieving nutritional requirements [32] and prevents under-consumption because of boredom effects and sensory specific satiety [33]–[35]. Subjects showed a clear preference for savoury over sweet food items when increasing intake of ‘anytime’ foods on the 10% protein diet (even though both food types were of the same macronutrient composition). This may reflect habitual preferences or may be an indication of participants seeking protein due to associating savoury sensory qualities with protein. The increased energy intake was disproportionately attributable to eating more between main meals rather than during meal times implying that protein influences energy intake through hunger and meal initiation rather than satiation or meal termination. This interpretation is supported by our results showing a greater increase in hunger ratings in the second hour following a 10% protein breakfast on study day 4 than for the higher protein breakfasts.

The idea that protein influences energy intake through hunger would also help to explain results from studies showing that mandatory high protein snacks are energetically compensated for at subsequent *ad libitum* meals but do not reduce total energy intake over a day [36], [37], and that eating a high-protein snack prolongs the time until a subsequent request for dinner [38]. Interestingly, the mean hourly hunger levels of participants across the entire day in the current study did not differ between dietary treatments. Similarly, Weigle *et al.* [18] found increased satiety ratings when participants were fed an iso-energetic 30% protein diet in comparison to 15% protein, but when the same participants were allowed to eat the 30% protein diet ad libitum, energy intake was decreased but satiety ratings were similar to those measured on the isocaloric 15% protein diet. This discrepancy between appetite scores and objectively measured hunger is not uncommon. A more detailed time-course of changes in hunger and fullness throughout the period from the end of a meal until the next *ad libitum* feeding episode, in a study design where participants initiate all meal times, may be more instructive in explaining patterns of intake in response to altered levels of dietary protein intake. Alternatively, changes in hunger and fullness may only be evident when high and low levels of protein intake are prescribed [18], [39] but not under *ad libitum* and constant food availability conditions [18]. In the latter circumstance, the participant may immediately respond to a small increase in hunger by eating prior to detection of the increase on a visual analogue scale. The mechanism of protein appetite cues is yet to be determined, but potentially involves the detection of reductions in intestinal and/or circulating free amino acid levels [40]–[42] and associated hormonal signals [43], [44].

Over the 4-day study periods, for every 1 kJ decrease in protein intake below the 15% level, non-protein intake increased by 4.5 kJ; whereas for every 1 kJ increase in protein intake above the target, participants decreased non-protein intake by 1 kJ. These results suggest an asymmetry in protein leveraging in humans, as inferred from earlier human studies and described experimentally in other animal models [2], [15], [45]. A general asymmetry of appetite is accepted [46]. This asymmetry may reflect the fact that the evolutionary costs of eating too little protein exceed those of eating too much [45], although excess protein consumption has also been shown to have associated costs in some animals [47] and perhaps in humans [48]. Nevertheless, reduced energy intake on high percent protein diets has been reported previously in studies in which macronutrient composition was not disguised [2], [7], [18]–[21]. Typically such studies have used higher protein (commonly 30%) dietary regimens, in overweight and obese individuals indicating a need for future protein leverage testing in such individuals. Alternatively the failure to adjust total energy intake on the 25% protein regimen in lean humans in the current study may have arisen from the constant availability and high level of variety of the study foods. We therefore suggest that high levels of food availability and variety may enable over-consumption on lower percent protein diets, promoting the chances that protein requirements will be met but also attenuating reductions in energy intake on higher percent protein diets that would otherwise arise via protein-dependent feedbacks. If we had incorporated higher fat levels into the study foods, the effect of protein leverage on energy intake by snacking may well have been substantially greater, given that fat has twice the energy density of carbohydrate and appears to provide significantly less suppression of appetite than carbohydrate [49].

As a result of failure to decrease intake on the 25% protein treatment, habitual protein intakes were exceeded on this treatment, unlike in the lower-protein treatments. As well as engendering possible health costs [48], physiological adaptation to higher protein intakes would be predicted to lead to an increase in the protein intake target [50], [51]. Having a higher protein target will, in turn, increase the susceptibility to overeat on a low percent protein diet since more total energy has to be ingested to achieve a higher target level. This has been proposed as a possible reason why oceanic populations appear more susceptible to overeating on a low percent protein western diet than populations that went through the agricultural revolution and have adapted to a lower percent protein in the diet [2], [15]. These populations may also be quite ‘thrifty’, with efficient storage but limited thermogenic capacities, further increasing the risk of obesity through overconsumption [27].

In the present study, we used carbohydrate as the diluent for protein in the diet, raising the possibility that the effects we observed were due to carbohydrate rather than protein. Rodents regulate intake of both protein and carbohydrate when provided with complementary foods, but when forced to trade-off overeating one macronutrient against undereating the other relative to this target mixture, protein dominates [11], [52]. If the same applies for humans, regulatory feedbacks for carbohydrate would have mitigated the response to dietary protein, with higher levels of carbohydrate in the 10% protein diet impeding increased consumption, and lower levels of carbohydrate on the 25% protein offsetting negative feedbacks from protein. Hence, although carbohydrate may have dampened the protein leverage response, it seems unlikely that it provides an explanation for the observed changes in energy intake that were maximal when subjects were consuming foods with high percent carbohydrate content. Neither is there evidence that other differences among the diets, whether associated with the foods themselves, or ‘self-dosed’ as a result of subjects eating more of the 10% protein diet, could have caused the increased consumption. For example, there was no association between total energy intake and the intakes of fibre, salt and sugar (separate from total carbohydrate). Future studies should systematically explore the interactions between protein, fat and carbohydrate, as well as other factors such as energy density, protein quality, glycemic index and fibre content.

It follows from our results that any change in the nutritional environment that encourages dilution of dietary protein with fat and/or carbohydrate will promote increased total energy intake and thus increase the risk that obesity might develop. Many sources of such encouragement exist in the modern westernised environment. Some are economic - fat and carbohydrate are cheaper than protein [15]; others reflect an increasing reliance on processed foods which are often higher in fat and refined carbohydrate than unprocessed foods, and yet other influences include our evolutionary heritage, which has left us with a predilection for foods with a high fat and sugar content [16], [17]. To make matters worse, it appears that the beneficial side of protein leverage – reduced intake on high percent protein diets – may be diminished in westernised countries in which the variety and availability of foods, especially snack foods, is greater than it has ever been in our evolutionary history.

## Methods

### Ethics Statement

The study was approved by Sydney South West Area Health Service (Royal Prince Alfred Hospital) Human Research Ethics Committee (Protocol No. X07-0044) and the University of Sydney Human Research Ethics Committee (Ref No. 10153).

### Study participants

Lean, healthy (BMI: 18–25 kg/m^2^) male and female participants were recruited via advertising through casual employment sites at five universities within the Sydney region. The study was also advertised in local newspapers but this form of recruitment was not successful because of the time-commitment required for the study. Exclusion criteria included diabetes, high blood pressure, gastrointestinal problems, asthma, eczema or hay fever, chronic medical conditions, anaemia, allergies or strong dislikes to any study foods, smoking, following a weight reducing diet within the 3 months prior to the screening interview, pregnancy and breastfeeding. Participants completed the EAT-26 questionnaire and were excluded if they had a history of eating disorders or irregular eating habits. Vegetarians and vegans were excluded to aid in preparation of the treatment foods. 53 females and 28 males attended a screening interview. 22 females and 12 males were eligible, indicated their willingness to undertake the trial and completed initial investigation day measures. From these, 20 females and 10 males commenced the trial. Subsequently, three females chose to discontinue due to interference with university studies, illness or difficulties with blood collection. One male was excluded after commencement following a diagnosis of hyperthyroidism. Overall, 17 lean female and 9 lean male participants completed the trial, these were 24.3±1.3 (mean ± SEM; range 18–51) years of age and had a mean BMI of 21.8±0.4 (18–25.5) kg m^−2^. On completion, four participants were excluded from the data analysis for reasons including gastrointestinal upset during one of the study weeks (n = 1 female) and failure of one sub-group to comply with study procedures (n = 3 males, who ate one another's food). 16 lean female and 6 lean male participants were included in the final data analysis. These were 24.7±1.4 (mean ± SEM; range 18–51) years of age and had a mean BMI of 21.8±0.4 (18–25) kg m^−2^. All participants were given detailed verbal and written information regarding the purpose of the trial and the study procedures. All participants provided written consent. Participants were paid a AUD 100 instalment upon completion of each 4-day study period and a final payment of AUD 500 totalling AUD 800 for completion of all three study periods.

### Diet manipulation and final menu

The design, manipulation and testing of the foods used are presented in detail elsewhere 22. Briefly, recipes were modified to contain 10, 15 or 25% energy as protein. Carbohydrate was adjusted to be 60, 55 or 45% energy and dietary fat was kept constant at 30%. Energy density (kJ/g) was similar between the 10%, 15% and 25% versions of a given food item but was different between foods. These foods were tested prior to the start of the current trial where a separate group of lean, healthy subjects were presented with the 10%, 15% and 25% protein versions of each of the food items simultaneously. Participants sampled each version and then completed questionnaires testing for differences in pleasantness, sensory attributes and nutritional perception.

**Figure 4:**
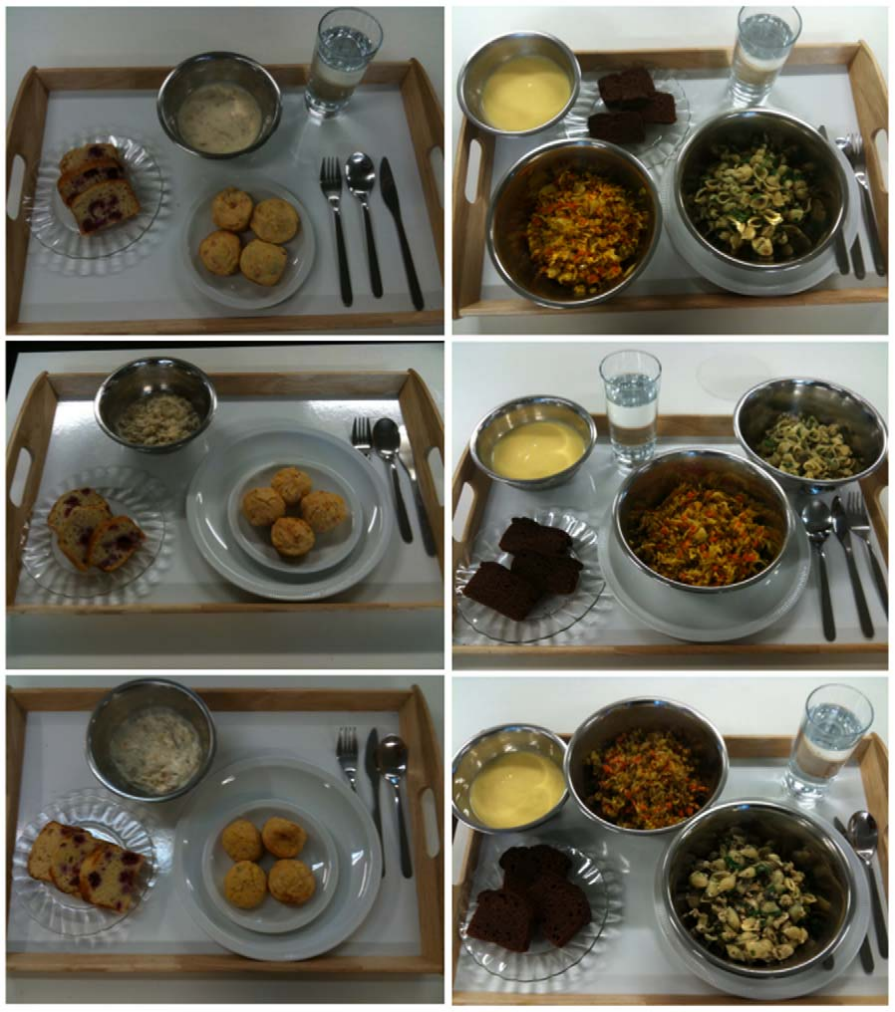
Protein leverage study foods. The 3 photos on the left column are the 10%, 15% and 25% versions (top to bottom) of each food given to participants at breakfast on study day 2. In the right hand column the three photos are the 10%, 15% and 25% versions (top to bottom) of each food given to participants at dinner on study day 2. Participants were offered a set amount of each food that was the same on each study period. The plates were the same for a particular food on each study period.

The final *ad libitum* menu, for each of the three, 4-day study periods contained the same 28 food items, including 12 sweet and 16 savoury foods (see Table 6 for full 4-day menu). In one of the study periods all the 28 foods contained 10% protein, in another period they all contained 15% protein, and in the third study period they all contained 25% protein. Menus were matched for energy density and palatability [22] (see Table 7 for nutritional information). Up to 12 foods were provided on each day during the 4-day period, giving participants both variety and choice at all times. Some foods were only available in the one meal sitting (‘meal time foods’), whereas others were available to participants anytime once served (‘anytime foods’). Anytime foods were ‘snack’ foods and foods that were first served at a meal and could be kept if not eaten or finished at that meal (Table 6). Therefore, between meals a participant had up to 4 sweet and 2 savoury options from which to choose. The foods were served as specific amounts. Foods served at meals were presented to each participant on a tray (Figure 4). ‘Anytime’ foods were labelled for identification and stored in a refrigerator, to which the participants had free access at all times. A variety of plates were used to present the different foods, but the 10, 15 and 25% versions of a particular food were always presented on the same style of plate in all three study periods (Figure 4).

**Table 6:** 4-day *ad libitum* menu for the 10%, 15% and 25% protein study periods.

	Study day 1	Study day 2	Study day 3	Study day 4
**Breakfast**		**Savoury breakfast muffin**	**Savoury breakfast muffin**	**Savoury breakfast muffin**
8.30–10.00am				
		Apricot yoghurt muesli	Raspberry yoghurt muesli	Apricot yoghurt muesli
		**Pear, raspberry & coconut bread**	**Banana bread**	**Pear, raspberry & coconut bread**
**Lunch**	Tuna bake	Mexican wrap	Tandoori wrap	Sweet potato wrap
1pm				
	Beef and vegetable pastry	Teriyaki sushi roll	Beef and vegetable pastry	Pasta salad
	Salad & dressing	Salad & dressing	Salad & dressing	
	**Fruit salad yoghurt**	**Apple crumble muffins**	**Fruit salad yoghurt**	**Apple crumble muffins**
**Dinner**	Goulash	Mushroom Pasta	Pasta Bolognaise	Hokkien noodles
6.30pm				
	**Cheese Scones**	Chow mein mince	**Cheese Scones**	Massaman curry
	Salad & dressing		Salad & dressing	
	**Orange & poppyseed cake**	**Chocolate, apple & ricotta cake**	**Orange & poppyseed cake**	**Chocolate, apple & ricotta cake**
	**Custard**	**Custard**	**Custard**	**Custard**
**Snacks**	**Savoury scones**	**Cheese scones**	**Savoury scones**	**Cheese scones**
all day				
	**Carrot cake**	**Raspberry yoghurt**	**Apricot muffins**	**Raspberry yoghurt**

Foods offered during the 10%, 15% and 25% protein 4-day *ad libitum* study periods. The methodology used to design each of these foods and the final nutritional information can be found elsewhere [22]. Some foods were only available in one meal sitting (‘meal time foods’: not bold), whereas others were available to participants anytime once served (‘anytime foods’: bold). Anytime foods were ‘snack’ foods and foods that were first served at a meal and could be kept if not eaten or finished at that meal; these foods were labelled for identification and kept in a refrigerator to which participants had free access.

**Table 7:** Total nutrition and weight of food available to participants over the 4-day *ad libitum* periods.

	energy	food	protein	fat	carbohydrate	fibre	sugars	sodium
	MJ/4 day	weight kg/4day	MJ/4 day	MJ/4 day	MJ/4 day	g/4day	g/4day	mg/4day
**10%**	84.720	12.550	8.510	24.950	51.010	195	874	26534
**15%**	84.560	12.590	12.350	25.140	46.830	187	1026	29034
**25%**	84.750	12.850	20.680	25.290	38.540	169	1040	31632

**Figure 5:**
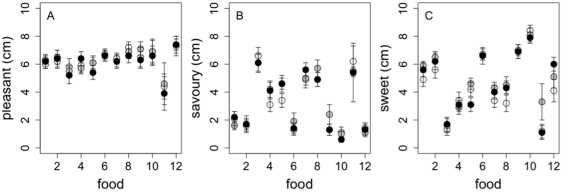
Palatability of foods served on each 4-day *ad libitum* period. Pleasantness (A), savouriness (B) and sweetness (C) ratings given to the 10% (white), 15% (grey) and 25% (black) versions for the 12 foods offered on study day 4: 1. Apricot, walnut and yoghurt muesli, 2. Pear, Raspberry and Coconut Bread, 3. Savoury Breakfast Muffins, 4. Pasta Salad, 5. Sweet Potato and Ricotta Wrap, 6. Apple Crumble Muffins, 7. Hokkien Noodles, 8. Massaman Curry, 9. Chocolate, Apple and Ricotta Cake, 10. Custard, using visual analogue scales scored from 0 to 10 cm.

The nutritional information for each 4-day *ad libitum* menu (10, 15 and 25% protein) is presented in Table 7. To confirm that palatability was similar between the 10, 15 and 25% protein versions of each food, food palatability was tested on study day 4 of each experimental period using a 10 cm visual analogue scale. All 12 foods offered to participants on study day 4 of each study period were rated for pleasantness, sweetness and savouriness and confirmed the absence of differences (Figure 5A–5C) as observed for other subjects in pre-trial testing [22]. Participants were offered optional un-manipulated foods including 10 g salad and 2 types of low-calorie dressings (10 g of each) with most meals. In addition, participants were asked to consume 150 g of skim milk per day to drink with tea, herbal teas and/or decaffeinated coffee.

### Study design

Each participant attended three 4-day periods of in-house dietary manipulation at the Woolcock Institute Sleep Study Centre, each separated by at least 1 week. In order for habitual energy and protein intakes to be estimated, subjects arrived for each three 4-day period with a completed 4-day food diary and a 24-h urine collection for measurement of urea excretion (Figure S2). Subjects were tested in single-sex groups of 2–3 and each group remained together throughout the full experiment. During each 4-day study period participants were provided with *ad libitum* food comprising 10%, 15% or 25% protein, so that by the end of the experiment each group had undergone 4 continuous days of each of the 10%, 15% and 25% menus. Only the participants were blinded to the treatment. Breakfast was provided between 8–10am, lunch at 1pm and dinner at 6.30pm and snacks were freely available at all times. If participants had not attended breakfast by 10am they were woken. Participants were taken for a 1-hour supervised walk each day. On day 4 participants were asked to complete visual analogue scale questionnaire for the measurement of palatability of each of the study foods tasted that day and also subjects' subjective hunger and to complete a 24-h urine collection for measurement of urea excretion.

### Measurement and validation of habitual protein intake

Four-day food diaries completed immediately prior to each 4-day *ad libitum* study period were analysed for daily protein and total energy intakes using Foodworks 2007. A 24-h urine collection was completed on the fourth day of the 4-day food diary, as well as on day 4 of each study period. The volume of urine and urinary urea concentrations were measured to calculate 24-h urine urea excretion. A highly significant correlation between 24-h protein intake and 24-h urine urea excretion was found (t_50_ = 15.7, p<0.0001) (Figure S2). Habitual protein and urea intakes fit this line (Figure S2), validating habitual protein intakes estimated from food diary analysis.

### Measurement of food intake

Participants were given *ad libitum* access to study food with no access to other food sources during each experimental period. Food intake was measured by recording the weight of the food before and after serving, to the nearest gram. Energy intake was then calculated using the nutritional information for each recipe [22].

### Subjective hunger and fullness

Visual analogue scales are a validated method to test appetite levels [53]. On study day 4, subjects completed a visual analogue scale (VAS) questionnaire to assess subjective hunger and fullness. The VAS questionnaire asks subjects to assess their hunger by reference to a 10 cm horizontal line anchored at one end with the extreme feeling “not at all hungry” and at the other end with “very hungry” and fullness with the extreme feeling “not at all full” and at the other end “very full”. This was done hourly from before breakfast until 22:00. The mean hunger scores and the lowest and highest values between breakfast and 22:00 were calculated. The score before breakfast and within the first and second hour following breakfast were averaged to determine if there was an effect of percent protein on hunger and fullness from a baseline meal. The change in hunger and fullness that occurred in the first and second hours following breakfast were calculated by difference between the score before breakfast and 1 hour after breakfast and between the score 1 hour and 2 hours after breakfast. From 12:00 onwards, hourly scores were plotted. Area under the curve was not measured due to missing values occurring when participants had a sleep during the day.

### Statistical Analysis

All data analysis and graphics were performed using *R* software [54]. The data are routinely presented as means ± SEM. Nutrient intake data and subjective hunger and fullness scores and the change in hunger and fullness following breakfast were analysed using one-way within-subject ANOVA. The data were checked for sphericity using Mauchlys sphericity test (*Multcomp* package [55]). If the sphericity assumption was violated, Greenhouse-Geiser corrections were applied to the F- and p-values and are indicated by the additions of ‘*’. Post-hoc analysis was performed with pair-wise comparisons using the Bonferroni correction for multiple comparisons.

## Supporting Information

Figure S1 **Total protein and non-protein intake for individuals over each 10%, 15% and 25% protein 4-day** ***ad libitum*** **study periods.** Bi-coordinate intake plots for individual subjects on 10, 15 and 25% protein 4-day treatment periods (dashed lines, females; solid lines, males). The range of total energy intakes (the sums of the x- and y- coordinates) on the 15% protein treatment period was 0.8 times the Schofield equation estimate (subject 15) to 2.1 times (subject 2), with a mean of 1.55±0.1.
(TIFF)

Figure S2 **Estimation of habitual protein intake and percent dietary protein.** Bi-coordinate means for 24-h protein intake (MJ) versus 24-h urine urea excretion (moles) for participants during the 4-day 10% (white triangle), 15% (grey triangle) and 25% (black triangle) *ad libitum* study periods. The dashed line represents the positive linear regression between 24-h dietary protein intake and total urine urea excretion (t_(50)_ = 15.7, p<0.0001). Average daily habitual protein intake and 24-h urine urea excretion prior to each study 10% (white circle), 15% (grey circle) and 25% (black circle) *ad libitum* study periods are also added to the plot.
(TIFF)
