# Supplementary figures and images for: Testing Protein Leverage in Lean Humans: A Randomised Controlled Experimental Study
Source: PLoS One. 2011 Oct 12;6(10):e25929. doi: 10.1371/journal.pone.0025929 (PMC3192127; doi:10.1371/journal.pone.0025929)

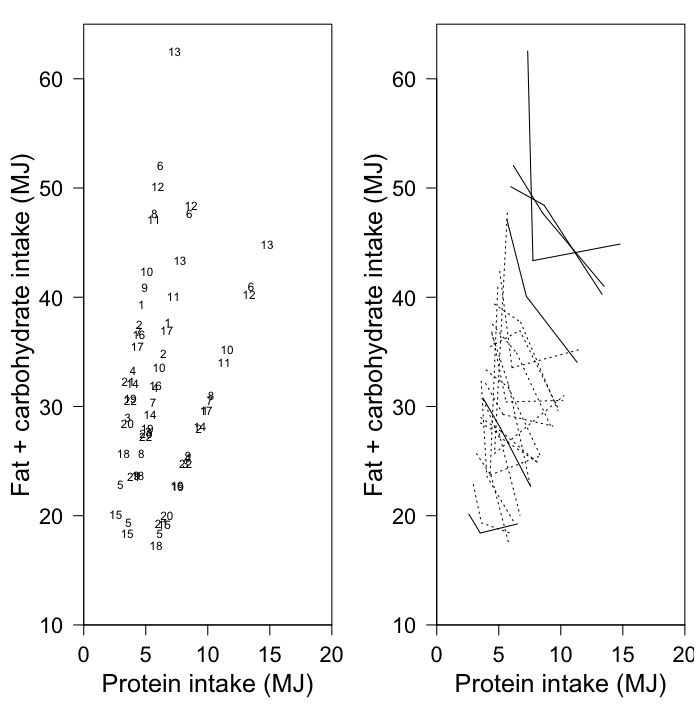

Supplement: Figure S1 — Total protein and non-protein intake for individuals over each 10%, 15% and 25% protein 4-day ad libitum study periods. Bi-coordinate intake plots for individual subjects on 10, 15 and 25% protein 4-day treatment periods (dashed lines, females; solid lines, males). The range of total energy intakes (the sums of the x- and y- coordinates) on the 15% protein treatment period was 0.8 times the Schofield equation estimate (subject 15) to 2.1 times (subject 2), with a mean of 1.55±0.1. (TIFF) [file pone.0025929.s001.tiff]

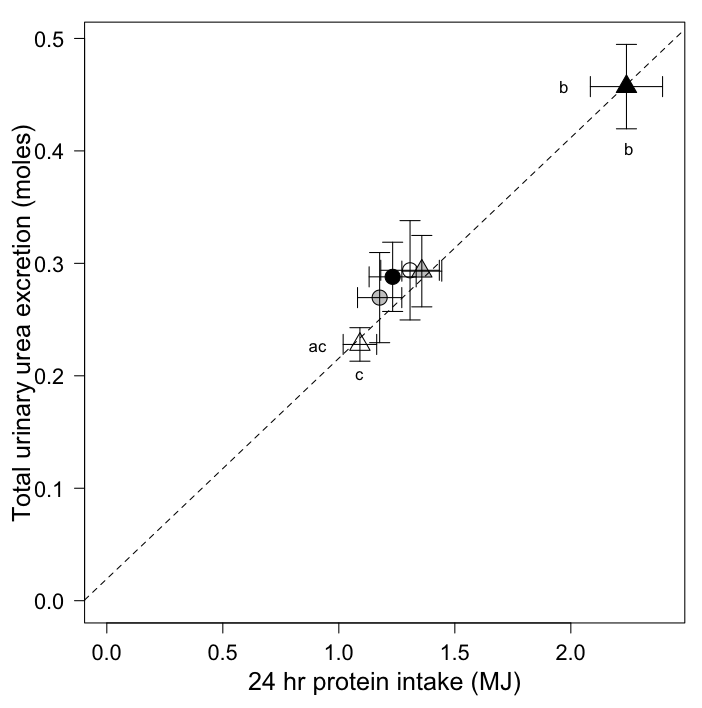

Supplement: Figure S2 — Estimation of habitual protein intake and percent dietary protein. Bi-coordinate means for 24-h protein intake (MJ) versus 24-h urine urea excretion (moles) for participants during the 4-day 10% (white triangle), 15% (grey triangle) and 25% (black triangle) ad libitum study periods. The dashed line represents the positive linear regression between 24-h dietary protein intake and total urine urea excretion (t(50) = 15.7, p<0.0001). Average daily habitual protein intake and 24-h urine urea excretion prior to each study 10% (white circle), 15% (grey circle) and 25% (black circle) ad libitum study periods are also added to the plot. (TIFF) [file pone.0025929.s002.tiff]
